# Neuroinflammation and Neurogenesis in Alzheimer’s Disease and Potential Therapeutic Approaches

**DOI:** 10.3390/ijms21030701

**Published:** 2020-01-21

**Authors:** Pi-Shan Sung, Po-Yu Lin, Chi-Hung Liu, Hui-Chen Su, Kuen-Jer Tsai

**Affiliations:** 1 Department of Neurology, National Cheng Kung University Hospital, College of Medicine, National Cheng Kung University, Tainan 704, Taiwan; pishansung@gmail.com (P.-S.S.); pylin1991@gmail.com (P.-Y.L.); shjmirage@yahoo.com.tw (H.-C.S.); 2 Institute of Clinical Medicine, College of Medicine, National Cheng Kung University, Tainan 704, Taiwan; 3 Department of Neurology, Linkou Chang Gung Memorial Hospital, Taoyuan 333, Taiwan; ivanliu001@cgmh.org.tw; 4 College of Medicine, Chang Gung University, Taoyuan 333, Taiwan; 5 Research Center of Clinical Medicine, National Cheng Kung University Hospital, College of Medicine, National Cheng Kung University, Tainan 704, Taiwan

**Keywords:** Alzheimer’s disease, neurogenesis, neuroinflammation

## Abstract

In adult brain, new neurons are generated throughout adulthood in the subventricular zone and the dentate gyrus; this process is commonly known as adult neurogenesis. The regulation or modulation of adult neurogenesis includes various intrinsic pathways (signal transduction pathway and epigenetic or genetic modulation pathways) or extrinsic pathways (metabolic growth factor modulation, vascular, and immune system pathways). Altered neurogenesis has been identified in Alzheimer’s disease (AD), in both human AD brains and AD rodent models. The exact mechanism of the dysregulation of adult neurogenesis in AD has not been completely elucidated. However, neuroinflammation has been demonstrated to alter adult neurogenesis. The presence of various inflammatory components, such as immune cells, cytokines, or chemokines, plays a role in regulating the survival, proliferation, and maturation of neural stem cells. Neuroinflammation has also been considered as a hallmark neuropathological feature of AD. In this review, we summarize current, state-of-the art perspectives on adult neurogenesis, neuroinflammation, and the relationship between these two phenomena in AD. Furthermore, we discuss the potential therapeutic approaches, focusing on the anti-inflammatory and proneurogenic interventions that have been reported in this field.

## 1. Introduction

Alzheimer’s disease (AD) is the most common neurodegenerative dementia worldwide and is characterized by early impairment of recent memory. As the disease severity progresses, AD patients have extensive symptoms, including impaired language, orientation, and executive functions, which lead to a decline in self-care ability. The two hallmark pathologies of AD are β-amyloid (Aβ) deposition and neurofibrillary tangles (NFTs) containing hyperphosphorylated tau (P-tau). A definitive diagnosis of AD requires an autopsy of brain tissues. However, recent emerging evidence has proposed that biomarkers of cerebrospinal fluid (CSF) (Aβ_40_, Aβ_42_ or its ratio and total tau or hyperphosphorylated tau in CSF) and positron emission tomography (amyloid-PET or tau-PET) combined with clinical criteria could assist in diagnosing living people [1].

The disruption concerning Aβ is the result of an overproduction or reduced clearance [2]. In addition, aging, as well as genetic or environmental factors contribute to the metabolic shift of amyloid precursor protein (APP) toward the amyloidogenic processing pathway [3]. Aβ peptides aggregate into insoluble oligomers and protofibrils, further instigating fibrillary Aβ species and, then, accumulate into senile and neuritic plaques. In addition to overproduction of Aβ, a reduction in Aβ clearance from the brain also results in the extracellular accumulation of Aβ and, subsequently, cascades of P-tau deposition, cytoskeletal changes, neuronal dysfunction, and neuronal death develop [4]. However, the physiological function of APP is still unknown and Aβ plaque has been observed 10 years or more before the onset of observable AD symptoms or a diagnosis [5].

Tau protein, a microtubule-associated protein, stabilizes microtubules. While tau protein is abnormally hyperphosphorylated and glycosylated, its biological function is impaired in AD. Abnormal folding of P-tau leads to the generation of paired helical filaments (PHFs) and NFTs, which are additional key neuropathological features of AD [6,7]. PHFs induce intracellular accumulation of tau protein in aggresomes [6] and the release of PHF from neurons into extracellular spaces induces the propagation of tau pathology across the different brain regions in AD patients [8,9]. The severity of tau accumulation is closely associated with dementia severity and neurodegenerative processes in AD [10,11].

Although a number of studies have indicated that amyloid deposition and tau proteins are two core neuropathologies responsible for AD, results of clinical trials that modulated these two pathological proteins by means of straightforward amyloid- or tau-immunotherapy were disappointing [12,13,14,15]. There is still a considerable gap in our understanding of AD pathogenesis. For example, the brain Aβ level increases in many neurological conditions other than AD, such as traumatic brain injury, stroke, multiple sclerosis, amyotrophic lateral sclerosis, and hypoxic ischemic encephalopathy. The mechanism for the increase in the Aβ level is still unclear but it could be a response to a neuronal insult or damage. Thus, in AD, the overproduction or accumulation of Aβ could be an attempt to repair the initial neuronal injury and maintain normal brain function [16]. The upstream pathophysiological process is neuroinflammation or abnormal microglia activation [17,18,19,20]; metabolic failure, regardless of etiology [21]; oxidative stress [20]; or sustained cholesterol-associated neuronal distress [22]. In addition, the recent “stress threshold change of state” model has been proposed to explain the presence of early-onset AD and late-onset sporadic AD [23]. To date, a considerable gap regarding the onset and progression of AD neuropathology and clinical symptoms still exists.

Over the last few decades, a third core neuropathological feature of AD has emerged. In addition to Aβ and NFT, neuroinflammation is exhibited in the brains of AD patients [18,19,24,25,26,27,28,29]. Such neuroinflammatory changes in AD have been observed in animal models [30,31], postmortem human brains [32,33,34], and through molecular imaging to detect inflammatory processes [35,36]. A number of studies have discussed the potential role of microglial genes in the regulation of neuroinflammation and the relationship between microglial genes and late-onset sporadic AD [29,37,38,39,40,41]. This neuroinflammatory process is responsive to neuronal loss or to the presence of pathological protein aggregation in AD. However, a number of studies have also suggested that sustained neuroinflammation is noted in the early disease phase of AD. With the progression of disease, such early inflammation facilitates and exacerbate the generation of Aβ and NFT and further results in neuronal toxicity and death [24,42,43,44,45].

Neurogenesis occurs through the division of neural stem cells (NSCs), the maturation of neural progenitor cells (NPCs), and then, migration and maturation into neurons [46]. The process was thought to halt postnatally, but has unexpectedly been detected in adulthood, even throughout the lifetime of the mammalian brain [47]. Adult neurogenesis in the mammalian brain has only been detected in certain limited regions, i.e., the subventricular zone (SVZ) and the subgranular zone (SGZ) of the hippocampus [48]. These regions maintain a neurogenic stem cell niche. Subventricular neurogenesis contributes to olfaction and olfactory neural circuitry [49]. In the SGZ, mature granular cells pass through multiple developmental stages. Stage one (proliferation) is manifested by NSCs, or type 1 radial glial-like cells that express glial fibrillary acidic protein (GFAP), nestin, and sex-determining region Y-box 2 (Sox2). Stage two (differentiation) is represented by intermediate progenitor cells (type 2 cells) that express doublecortin (DCX) or polysialylated neural cell adhesion molecule (PSA-NCAM). Then, type 2 cells give rise to stage three (migration) neuroblasts (type 3 cells) or neuronal lineage committed cells that express DCX, PSA-NCAM, and markers for immature neurons (Tuj-1b or NeuroD). Stage four (axonal and dendritic targeting) and stage five (synaptic integration) are manifested by mature neurons that express calretinin, as well as NeuN in stage four and calbindin in stage five, respectively [50]. Adult hippocampal neurogenesis (AHN) plays a crucial role in various hippocampus-dependent functions, including learning and mood [51,52,53,54]. Altered or dysregulated adult neurogenesis has been found in various neurodegenerative disorders, such as Parkinson’s disease, AD, or Huntington’s disease [54,55,56,57,58]. Previous studies have shown that genetic, environmental, and pharmacological factors play a role in regulating AHN [46,55]. Inflammation, either the systemic inflammation or neuroinflammation commonly noted in neurodegenerative disorders, also have a relevant influence on AHN [49,54,59]. Due to the interconnection between neuroinflammation and neurodegeneration in AD, in this review, we summarize the current evidence regarding the impact of neuroinflammation on AHN and its relevance to AD. In addition, we discuss the potential therapeutic strategies regarding this issue.

## 2. Adult Neurogenesis and Its Alteration in AD

### 2.1. Neurogenesis in Adult Human Brains

The concept of adult neurogenesis in humans was first published in 1998 by examining bromodeoxyuridine (BrdU)+ cells in the postmortem tissue from cancer patients [60]. In 2010, evidence of neurogenesis was reported using the technique of immunohistochemistry to identify the neurogenesis markers in postmortem human brain tissue samples [61]. The discovery of rostral migratory stream (RMS) reported by Sanai et al. [62] suggested the rudimentary continued olfactory neurogenesis. Although the presence of RMS is active in fetal age of human brain, it remains controversial in the adult brain [50]. Debates also existed due to contradictory findings of detectable hippocampal neurogenesis in adult human brains. A report noted that young neurons were detected in only the first year of life. Then, the recruitment of young neurons did not continue in the following years and was even extremely rare in the adult brain [63]. However, there is growing evidence supporting the presence of AHN in humans [47,64]. Moreno-Jimeńezetal et al. noted that the recruitment of new neurons can still occur and be detected in adulthood. With the shorter postmortem times and well-characterized fixation protocols, thousands of new neurons or immature neurons were detected in the dentate gyrus in neurologically healthy human subjects between middle age and the ninth decade of their life [47]. Improvements in study protocols and tissue preservation techniques have helped to detect the recruitment of new neurons in the human brain, therefore, supporting the presence of AHN in adulthood.

Although the evidence may not be sufficient to completely characterize the role of AHN, most researchers suggest that it can contribute to brain plasticity in adulthood. Potential functions of adult neurogenesis include increasing resilience against stress [65], improving pattern separation [66] and formation of memory and learning [67,68], and enhancing the loss of established or old memories [69]. Therefore, AHN plays a role in hippocampal plasticity [66,70], and the continuous addition of these new neurons in the dentate gyrus remodels the hippocampal circuits.

Increasing evidence has shown that many behavioral factors regulate adult neurogenesis. Running and enriched environmental stimulation can induce neurogenesis [71,72,73]. In contrast, chronic stress can suppress the proliferation of NSCs [74,75]. The amount of neurogenesis also depends on the individual’s age. During aging, NSCs and their progenitor cells reduced cellular proliferation and neuronal production [76,77,78]. A recent report found approximately 42,000 immature neurons/mm^2^ in the brain of a 43-year-old donor, but the number of immature neurons dropped by approximately 30% in the brain of the elder donor at the age of near 90 years [79]. Age-related neurodegenerative diseases, such as AD, have different impacts on NSC maintenance, proliferation, survival, and functional integration. Impaired neurogenesis in neurodegenerative disorders can cause the sustained loss of live neurons with a diminished renewal capacity. This can have an impact on the pathophysiological mechanisms of these neurodegenerative diseases.

### 2.2. Evidence Regarding Alteration in Adult Neurogenesis in AD

Adult neurogenesis has been studied in different transgenic animal models of AD with largely different experimental conditions, depending on the use of PSEN1, PSEN2, or different APP single mutation or the combinations. In addition, the regimens, doses, and the time points for analysis after BrdU treatment were also varied in these studies. However, under different conditions, altered and dysfunctional neurogenesis was noted in both the SVZ and SGZ of the dentate gyrus [80]. Such compromised neurogenesis can precede the onset of hallmark lesions and neuronal loss [80]. Mostly, adult neurogenesis is impaired in the AD transgenic model with a single mutation of PSEN1 [81,82,83]. APP transgenic single mutation (PDAPP) with the use of the PDGF promotor also negatively impacted on the proliferation and survival of NPCs, especially in the aged state after amyloid deposition [84]. However, double or triple mutations of APP (APP751swe, APPswe, Ind.) led to increased proliferation and differentiation of new neurons [85,86,87,88]. The increase in neurogenesis could be explained by a compensatory response to pathological protein accumulation [85]. Another report used eight-month-old APP/PS1 mice to show decreases in quiescent nestin-positive astrocyte-like stem cells with preserved numbers in transient amplifying progenitor cells. However, both cells exhibited morphological abnormalities during amyloid deposition [89]. These findings provide evidence that the amyloidogenic environment leads to NPC dysfunction and that AHN is altered differently in various animal models of AD. In addition to transgenic animal models of AD, intracerebroventricular (ICV) injections of a low and subdiabetogenic doses of streptozotocin (STZ) have been demonstrated to be a new nontransgenic animal model to mimic human sporadic AD [90]. ICV injection of STZ induced oxidative stress, neuroinflammation, cerebral cholinergic deficits, and cognitive deficits that mimic AD. In this nontransgenic AD animal model, an ICV injection of STZ negatively impacted the generation of immature and mature neurons in the long-term period (three months) [91].

Variable findings in adult neurogenesis were also observed in human AD brains. One earlier report demonstrated that AD brains had higher expression of DCX, PSA-NCAM, TOAD-64/Ulip/CRMP (TUC-4), and NeuroD than that in the brains of normal subjects, indicating increased neurogenesis in the human AD brains [85]. Another report exhibited an increased number of Ki-67+ cells in the CA1-3 region of presenile human AD brains, reflecting an increase in glial- and vasculature-associated changes, but no altered neurogenesis was revealed in the dentate gyrus [92]. However, contradictory data existed. Dramatically decreased expression of the mature neuronal marker high molecular weight microtubule-associated protein (MAP) isoforms MAP2a was found in the dentate gyrus of human AD brains, indicating a failure of neuronal maturation in the hippocampus [93]. Another study reported a decrease in DCX+ and Sox2+ cells in the dentate gyrus of AD hippocampus as compared with nondemented control cases [94]. Recently, Moreno-Jimeńezetal et al. [79] used tightly controlled conditions and state-of-the-art tissue processing methods to study AHN in 45 patients with AD between 52 and 97 years of age. The number of DCX+ cells markedly declined as the AD neuropathological stage advanced. In addition, the number of DCX+ cell was consistently lower in AD patients than in neurologically healthy controls, regardless of age. They found that AHN was altered during the early stages of AD, even in the Braak stage I or II, in which the NFT was confined in the transentorhinal region of the brain. These data provide evidence that the neurogenesis in AD differs from physiological age-related altered neurogenesis. Some independent pathophysiological mechanisms could contribute to impaired AHN in AD, even in the early stage of the disease.

### 2.3. Known Modulators of Adult Neurogenesis and the Effects of AD on Adult Neurogenesis

Multiple intrinsic and extrinsic factors have been reported to regulate adult neurogenesis. Known intrinsic modulators include signal transduction pathways (Wnt signaling, Notch signaling, Sonic hedgehog signaling (Shh), and Eph:ephrin signaling) [95,96,97,98,99,100,101,102]; epigenetic modulators, such as methyl-CpG-binding domains (MBD)-1 [103], methyl-CpG-binding protein 2 (MeCP2) [104], DNA-damage-inducible protein 45 beta (Gadd45b) [105], histone acetylation (HDAC3, HDAC5, and HDAC7) [106], and microRNAs (Let-7b, miR-9, miR-34a, and miR184) [106]; and genetic variation, such as RE-1 silencing transcription factor gene (REST) [107] and G-coupled protein receptor adenosine receptor A2A (ADORA2A) [108]. Extrinsic modulators include metabolic growth factors, such as VEFG, BDNF, IGF-1, FGF-2, IGF, and PDGF, which play a role in contributing to the proliferation, migration, cell fate determination, and maturation of NSCs or NPCs [109,110,111,112,113]. Another extrinsic modulator includes vasculature and angiogenesis, in which some reports suggested that the vascular beds of SVZ and SGZ support neurogenesis, and that endothelial cells promote the proliferation, differentiation, and survival of NSCs [114,115,116]. The immune system is another important modulator of adult neurogenesis. Various studies have demonstrated that inflammation affects adult neurogenesis by either proneurogenic effects or antineurogenic effects. Whether the effect enhances or inhibits depends on how to activate microglia, macrophages, or astrocytes and the duration of inflammation [117,118]. A previous study showed that resting microglia removed the apoptotic newborn neuroblasts by phagocytosis, indicating that microglia contribute to maintaining the homeostasis of the neurogenesis cascade in the dentate gyrus [119]. Another in vitro study also provided data that microglia have an instructive role for neuronal cell differentiation [120]. However, activated microglia, especially the classically activated proinflammatory (M1) phenotype, promote inflammation and play a role in suppressing hippocampal neurogenesis by reducing the survival of neuroblasts [121,122,123]. One landmark animal study, published in 2003, showed that peripheral injection of lipopolysaccharide (LPS) decreased the number of DCX+ cells and increased the number of microglia in the dentate gyrus. This LPS-induced attenuation of AHN was reversed by non-steroid anti-inflammatory drugs [122]. Moreover, long-term peripheral LPS injections for 28 days in adult mice had a long-term negative impact on AHN [121]. In addition to the effect of microglia and the duration of inflammation, some cytokines or chemokines, such as IL-1α, IL-6, IL-10, CX3CL1, CXCL1, and CXCL12 have important roles in positively regulating neurogenesis [124,125,126,127,128,129,130]. The proinflammatory cytokines released by activated microglia or astrocytes, such as IL-1β, TNF-α, IL-18, and IFN-γ, and some chemokines, such as CCL11, negatively impact the proliferation or differentiation of NSCs [131,132,133,134]. The duration of exposure of these cytokines or chemokines also has different effects on neurogenesis. For example, acute exposure of IL-6 cocultured with adult rat hippocampal NSCs for six to seven days significantly induced the differentiation of NSCs [124], but long-term exposure of the brain to IL-6 interfered with adult neurogenesis [135]. Briefly, neuroinflammation could be beneficial as a physiological response to maintain brain homeostasis but could also be detrimental to adult neurogenesis while it turns out to be a chronic process.

How AD affects adult neurogenesis is still a matter of debate, but a certain number of molecules central to AD have been noted to play a regulatory role in adult neurogenesis. PSEN1, a catalytic core of the aspartyl protease γ-secrease, regulates NPC differentiation in the adult brain through EGFR and Wnt/β-catenin signaling [136]. Downregulation of PSEN1 in hippocampal NPCs caused the morphological changes of decreased dendritic branching and dendritic spines and deficits in pattern separation and novelty exploration in behavioral tests [137]. One of the APP metabolites, soluble APP (sAPP), a cleavage product following APP cleavage by α-secretase, promoted neurite outgrowth in neurons differentiating from NPCs [138]. Other APP metabolites, such as sAPP-α, sAPP-β, Aβ peptides, and APP intracellular C-terminal domain (AICD) appear to modulate various functions in NSCs, including proliferation, neurogenesis, gliogenesis, or cell death [139]. Scopa, et al. [140] reported neurogenesis defects in a prodromal asymptomatic age (1.5 months of age) of TG2576 transgenic mice, in which the proliferation of NSCs was significantly reduced and the NSCs failed to differentiate into mature neurons. This reduction depended on the formation and accumulation of intracellular Aβ oligomers in NSCs, indicating the negative impact of Aβ oligomers on neurogenesis. AICD forms a transcriptionally active complex with Fe65 and Tip60 to regulate the expression of several genes, including PTCH1 (ptch1, receptor to suppress the Shh signaling in the absence of Shh ligands) [141] and Sox2 (transcription factor to regulate the embryonic development and cell fate determination) [142], and as previously discussed, plays a role in neurogenesis. Disintegrin-metalloproteinases (ADAMs) have α-secretase activity in vivo and are known for alpha site proteolysis of APP. ADAM10-knockout mice had a prominent decrease in the number of NPCs in the SGZ and the activation of Notch-1 and its downstream target gene was impaired in ADAM-deficient hippocampal tissue [143]. ADAM 17 processes many substrates, including Notch and the EGFR ligand, and are critical for multipolar exit and radial migration of neuronal intermediate progenitor cells [144]. ADAM 21 was shown to be expressed in the adult SVZ cells and was associated with the migration and differentiation of neuroblasts [145]. Hyperphosphorylation of tau protein has an impact on neurogenesis. In young APPswe/PS1ΔE9 mice, neurogenesis is impaired in the SVZ, and NPC proliferation and early differentiation are especially impaired [146]. In this area, the Aβ was relatively low, but hyperphosphorylation of tau was significantly increased as early as two months of age [80,146]. Moreover, glycogen synthase kinase 3β (GSK-3β), which is the main kinase that phosphorylates tau in AD [147,148], also affects neurogenesis. GSK-3β overactivation increases the apoptosis of mature neurons, impairs the differentiation of neuroblasts, and reduces the number of proliferative clusters within the dentate gyrus [149,150]. The alteration of GSK-3β activity in the dentate gyrus could underlie the alterations in tau phosphorylation in these neurogenic niches of the brains [150].

### 2.4. The Association between Altered Neurogenesis and AD Pathology

One may question the causal relationship between altered neurogenesis and AD complex pathology. The above-mentioned evidence showed that many pathological metabolites (e.g., APP metabolites) or key regulators involved in the AD process (e.g., PSEN1 or GSK-3β) play a role in influencing AHN. Is it possible that altered neurogenesis mediates AD pathologies? Choi et al. [151] recently used 5xFAD mice to study the relationship between neurogenesis and AD pathology. They use three models to block AHN by focal irradiation, DNA-ankylating agents (temozolomide, TMZ), and a lentivirus expressing a dominant-negative form of Wnt (LV-dnWnt). They found that a high degree of blocking AHN at a very early disease stage (six to eight weeks) did not affect the level of Aβ deposition or the gliosis severity, but initiated mature neuron death and exacerbate cognitive dysfunction in later disease stages (five months) in 5xFAD mice. Such a phenomenon was not noted in wild-type mice. This suggests that early alteration of neurogenesis exerts no influence on Aβ pathology, but impaired hippocampal neurogenesis in the early stage of AD could possibly increase hippocampal neuronal vulnerability, thus, leading to exacerbated cognitive impairment and increased neuronal loss in later stages of AD when the brain environment becomes more hostile.

## 3. Neuroinflammation and Its Impact on Adult Neurogenesis in AD

### 3.1. Neuroinflammation in AD

Neuroinflammation is the third core neuropathological feature in AD brains besides Aβ or NFT. Activated astrocytes and microglia are found characteristically around neurons and plaques. Increased expression of several proinflammatory cytokines or inflammatory markers was also noted in AD brains [152,153]. This inflammatory reaction was proposed to be a response toward the progressive accumulation of Aβ plaques and NFTs. Chronic or uncontrolled activation of these inflammatory processes is detrimental by inducing neuronal damage or death [154]. In the following section, we discuss many cellular components and mediators that constitute the neuroinflammation process in AD.

Microglia are the resident immune cells of the central nervous system. Similar to peripheral monocytes, they perform phagocytosis, antigen presenting, and produce immune mediators. In AD, microglia interact with Aβ oligomers and fibrils with scavenger receptors (SCARA-1, MARCO, SCARB-1, CD36, and RAGE), G-protein coupled receptors (FPR2 and CMKLR1), toll-like receptors (TLR2, TLR4, TLR6, and TLR9), CD47, α6β1 integrin, and TERM2 [18,155,156]. After initial recognition, microglia are activated through the NF-κB pathway [157]. Due to the persistent encounter of Aβ and the inefficient phagocytosis clearance of fibrillar form Aβ [158], microglia are persistently activated. Chronic activated microglia due to Aβ produces proinflammatory mediators, which lead to decreased phagocytosis ability and prolong neuroinflammation [159]. Astrocytes are multifunctional glial cells involved in nutritional supplementation to neurons, waste clearance, and blood–brain barrier maintenance. In AD, astrocyte activation [160], astrogliosis, and astrocyte atrophy [161], indicated by increasing GFAP, could occur early in the disease course, even before amyloid plaque formation. Aβ also activates astrocytes, possibly through the NF-κB pathway [162]. Activated astrocytes degrade Aβ itself and increase the phagocytosis of microglia through ApoE lipidation [163]. However, activated astrocytes also produce inflammatory mediators, contributing to the neuroinflammation process [164]. Oligodendrocytes are the source of myelin in the central nervous system. The role of oligodendrocytes in the neuroinflammation of AD is still largely unknown. In an in vitro study, oligodendrocytes are capable of complement synthesis, and thus could contribute to the neuroinflammation process [165].

Cytokines play a different role in neuroinflammation of AD. TNF-α increases Aβ generation form APP through β- and γ-secretase [166]. IL-1 increases the synthesis, secretion of APP, and generation of Aβ [166]. IL-1 also increases the phosphorylation of tau protein through the p38-MAPK pathway [167]. IL-6 increases the expression of APP [168], and also increases the phosphorylation of tau protein through the cdk5/p35 pathway [169]. Chemokines regulate microglial migration in the central nervous system. Aβ increased the level of CXCR8, CCL2, CCL3, and CCL4 production in human mononuclear cells [170]. CX3CR1/CX3CL1 is important for maintaining the microglial resting state maintenance and synapse maturation and function [171]. In an animal model, CX3CR1/CX3CL1 was shown to be related to amyloid deposition and cognitive decline [172]. The complement system components in the central nervous system are produced by microglia, astrocytes, oligodendrocytes, and neurons [173]. In AD, the role of the complement system remains to be clarified. C1q binds to Aβ and activates the alternative complement pathway, leading to enhanced phagocytosis of Aβ and neuroinflammation [174]. In an animal study, C3 deficiency was linked to impaired amyloid phagocytosis and increased Aβ deposition [175]. A genome-wide association study revealed an association between apolipoprotein J, a complement inhibitor, and complement receptor 1, further indicating the potential contribution of complement system to AD [176].

### 3.2. Evidence Regarding Neuroinflammation and Altered Neurogenesis in AD

Since neuroinflammation is an important hallmark of AD, it could have a remarkable influence on AHN in AD. The deposition of the Aβ protein activates microglia and astrocytes, and this activation is accompanied by the secretion of inflammatory cytokines, such as IL-1β, IL-6, and TNF-α. These cytokines have been proven to have a negative impact on AHN, including a negative effect on NSC proliferation and survival, as discussed previously. Although there is still no direct evidence regarding the impact of neuroinflammation on AHN in human AD brains, some studies using AD animal models have demonstrated the association between neuroinflammation and neurogenesis. Bassani et al. reported that an ICV injection of STZ (an animal model of AD) increased immunoreactivity of the glial markers Iba-1 and GFAP and decreased cell proliferation and the number of immature neurons (Ki-67+ and DCX+ cell) in the SVZ and dentate gyrus of the hippocampus [177]. Later, the same group used this rat model of AD to demonstrate that STZ caused an acute and persistent neuroinflammatory response in the SVZ and dorsal hippocampus, impaired short-term and long-term spatial memory, and reduced the survival, differentiation, and maturation of newborn neurons, which supports the hypothesis that neuroinflammation negatively affects neurogenesis [178]. In addition, Mishara et al. [179] reported that an ICV injection of STZ significantly reduced the number of BrdU+ cells, BrdU+/Nestin+ cells, and DCX+ cells in the SVZ and dentate gyrus, 11 and 18 days after the injection, indicating a negative influence on the proliferation of NSCs and migration of newborn neurons. A concurrent significant increase in the markers of neuroinflammation, including GFAP and NF-κB, was noted in the SVZ and hippocampus. This increase suggests an association between decreased neurogenesis and enhanced neuroinflammation in this kind of AD animal model. In the transgenic AD animal model, Kiyota et al. [127] used the adeno-associated virus (AAV) mediated gene delivery method to enhance the expression of the anti-inflammatory cytokine IL-10 in the hippocampus of APP/PS1 transgenic mice. The expression of IL-10 suppressed reactive gliosis and microglial accumulation around the plaque and enhanced neurogenesis by significantly increasing the number of BrdU+/NeuN+ cells and DCX+ cells in the SGZ of the hippocampus. Further in vitro microglia/NSCs coculture systems demonstrated that IL-10 enhanced the proliferation of NSCs by stimulating microglia, but not by IL-10 alone. This result supports the role of microglia in regulating the function of neurogenesis. In another animal model of AD, Ghosal et al. [180] reported that AICD transgenic mice (coexpressing the 59-residue long AICD fragment and Fe65, which form the transcriptionally active complex to regulate several genes involved in in the process of neurogenesis) had reduced numbers of BrdU+ cells and DCX+ cells in the SGZ at three months of age to at least 12 months of age, thus, demonstrating decreased hippocampal neurogenesis. They also found a dramatic recruitment of CD45+ microglia and increased expression of proinflammatory cytokines in 12-week-old AICD transgenic mice. An increase of BrdU+ and DCX+ cells in the SGZ was noted after oral treatment with the non-steroidal anti-inflammatory drugs (NSAIDs), ibuprofen and naproxen, for nine weeks, beginning at three weeks of age, thus, indicating the association between reducing neuroinflammation and enhancing neurogenesis in AD transgenic mice. Valero et al. [181] studied the effect of single systemic inflammation induced by an intraperitoneally LPS injection on adult neurogenesis in a wild-type and triple-transgenic mouse model of AD (3xTg-AD). A single, systemic LPS injection in 3xTg-AD mice at four months of age aggravated long-term hippocampal-dependent spatial memory impairments seven weeks after the LPS injection. The number of DCX+ cells in 3xTg-AD mice significantly decreased as compared with that in wild-type mice, and LPS injection also decreased the number of synaptic puncta in the dendrites of DCX+ cells of 3xTG-AD mice, indicating that a single systemic inflammatory event also produces a long-term impairment on the formation of synaptic specializations in new born neurons of AD transgenic animal models.

Although there is a lack of direct evidence regarding neuroinflammation and neurogenesis in human AD brains, the above-mentioned findings support the association between neuroinflammation and altered neurogenesis in AD models. Further studies may be warranted to verify the exact causal relationship between these two phenomena, especially in the degenerative process of AD. The findings of the above-mentioned changes between neuroinflammation and neurogenesis in AD animal models are summarized in Table 1.

## 4. Potential Therapeutic Approaches Targeting Adult Neurogenesis and Inflammation

It is unknown whether restoration of normal levels of adult neurogenesis becomes a potential therapeutic approach for slowing or reversing the progression of AD. Choi et al. [151] increased neurogenesis using a genetic method (mice received lentivirus expressing the WNT3 protein, LV-Wnt3, to increase the proliferation of NPCs) and a pharmacologic method (P7C3, a compound to enhance the survival of NPCs) in 5xFAD mice. They found that inducing neurogenesis alone had limited effects on improving cognition in AD transgenic mice. However, the method combined with pharmacologically increasing the level of BDNF (AMP-activated protein kinase agonist 5-aminoimidazole-4-carboxamide riboside) and neurogenesis activation by LV-Wnt3 or P7C3 ameliorated cognitive dysfunction in this AD model.

Dietary behavior, including caloric intake, meal frequency, meal texture, and meal content, has been reported to influence AHN [182]. The animal models showed that caloric restriction [183], hard-textured diet [184], and nutritional contents, such as polyunsaturated fatty acids (PUFA, such as omega-3 fatty acids) [185], polyphenols [186] (blueberries [187] and curcumin [188]), and vitamin D [189] positively regulate AHN and potentially restore behavioral impairment. Caloric restriction has neuroprotective roles in AD by increasing expression of neurogenesis-related genes and decreasing expression of inflammation-related genes in the PS1 and PS2 double knock-out mice [190]. Caloric restriction also enhances the expression of SIRT1, which is expressed in the hippocampus and is important for normal learning and memory. SIRT1 is involved in enhancing cell survival and neuronal differentiation [191,192]. In addition to enhancing neurogenesis, some nutritional contents also exert anti-inflammatory effects. PUFA exerted anti-inflammatory effects by affecting gene regulation involving inflammatory process in blood mononuclear leukocytes in AD patients [193]. Curcumin [194], quercetin [195], and caffeine/coffee [196,197] were shown to have anti-inflammatory roles, and therefore potentially beneficial for both AHN and protection against AD [198]. In addition to dietary intervention, probiotics, which are beneficial bacteria that modulate gut microbiota composition and function, have been shown to reduce neuroinflammation and restore cognitive deficits in AD animal model [199]. The supplementation of probiotics also increase BDNF mRNA in the hippocampus [200], thus potentially positively regulating AHN. However, the effect of probiotics supplementation on anti-inflammation or cognitive function in AD patients has not been very promising to date [201,202]. Future studies should focus on fields including proneurogenic and anti-inflammatory effects of dietary intervention and the modulation of gut microbiota and their therapeutic impact in AD models [198,203].

Another positive regulator of adult neurogenesis, environmental enrichment, has been demonstrated not only to reduce Aβ levels and amyloid deposition but also to reverse the impairment of adult neurogenesis and cognitive dysfunction in APPswe/PS1DE9 mice and APP23 mice [88,204]. However, negative results have been reported regarding the effect of environmental enrichment on adult neurogenesis in transgenic mice harboring FAD-linked PS1 mutations [83].

During the past decades, exercise has been demonstrated to exert repeated and robust beneficial effects on hippocampal neurogenesis. Praag et al. reported that voluntary running increased the proliferation and survival of new neurons in the dentate gyrus and long-term wheel running increased both hippocampal neurogenesis and the spatial learning abilities [205,206]. Such proneurogenic effects of running and improving performance of memory tasks have been demonstrated in different experimental paradigms in wild-type rodent models [207,208] and also in AD rodent models [151,209,210]. In human studies, there is a lack of direct evidence to reveal the relationship between exercise and enhancing neurogenesis. However, exercise has been shown to improve the memory tasks by different exercise training protocols in healthy populations [211] or diseased populations, such as patients with Parkinson’s disease [212] or AD [213].

Recently, some reports have suggested that voluntary exercise could have anti-inflammatory effects in the brain [214,215]. Animal studies have also proven that exercise restores the level of impaired neurogenesis induced by neuroinflammation [216]. However, there is as yet no direct evidence regarding the impact of exercise on neuroinflammation and neurogenesis in AD animal models. Parachikova et al. [217] reported that voluntary exercise in Tg2576 aged mice not only improved their spatial learning ability but also increased hippocampal expression of CXCL1 and CXCL12, which have been reported to have proneurogenic effects [129,130]. Nichol et al. [218,219] also reported that exercise reduced the expression of TNF-α and IL-1β in the hippocampus of aged Tg2576 mice. The use of an in-cage running wheel for three weeks also reduced the level of soluble Aβ and soluble fibrillary Aβ in transgenic mice. However, contradictory results have shown that exercise has no effect on modulating neuroinflammation [219]. Although some variant data have been reported, exercise could have a potential for AD-associated neuroinflammation and impaired neurogenesis. Future directions should focus on the proneurogenic and anti-inflammatory effects of exercise and its therapeutic impact in AD models.

Because neuroinflammation and their related cytokines or chemokines exert negative impacts on adult neurogenesis, some therapeutic approaches targeting inflammatory cells or inflammatory molecules to manipulate neurogenesis have been reported. In Ghosal’s report, impaired neurogenesis in AICD transgenic mice was prevented by oral treatment with buprofen and naproxen [180]. However, other studies focused on NSAIDs [220], or glucocorticoid steroids [221], in AD have not been very promising to date. Future studies are required to draw solid conclusions about the anti-inflammatory drugs on altered neurogenesis as a disease-modifying therapy for AD. 

**Figure 1:**
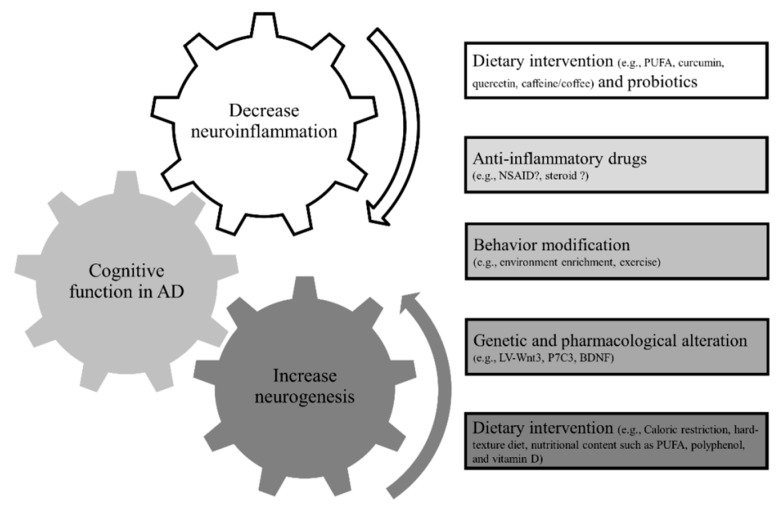
The potential therapeutic approaches targeting adult neurogenesis and inflammation in AD. PUFA, poly-unsaturated fatty acids; NSAID, nonsteroidal anti-inflammatory drugs; and LV-Wnt3, lentivirus expressing the WNT3 protein.

Figure 1 summarizes the potential therapeutic approaches listed in above section. 

## 5. Conclusions

Neurogenesis exists in aged human brain and its level is indeed decreased in human AD brain as compared with that in healthy controls. Animal models correspondingly supported that early impairment of neurogenesis could increase neuronal vulnerability in the hostile brain environment of AD. Inflammation is one of the central etiologies influencing the process of altered neurogenesis in AD. A better understanding regarding the interactions between altered neurogenesis and neuroinflammation in AD is required to develop a potential novel approach focused on modulating these two phenomena to ameliorate the neurodegenerative process of this disease.

## Figures and Tables

**Table 1:** The evidence regarding altered neurogenesis and inflammation or anti-inflammation in AD rodent models.

Reference	Model	Observation Period	Neurogenesis (Location)	Inflammation/Anti-Inflammation	Behavior
Bassani et al. (2017) [177]	ICV-STZ (Wistar rats)	4 weeks	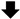 Ki-67+ 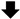 DCX+ (SVZ, DG)	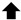 GFAP 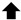 Iba-1 (SVZ, DG)	Impairment in short-term spatial memory (object location test and Y maze) and short-term recognition memory (object location test)
Bassani et al. (2018) [178]	ICV-STZ (Wistar rats)	Acute, 7 days Late, 30 days	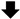 Ki-67+ cell (DG and SVZ) in acute period 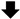 BrdU+NeuN+ cell (DG) in late period 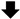 DCX+ cell (DG and SVZ) in late period	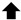 GFAP 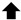 Iba-1 (CA1 and CA3, DG) in acute and late period	Impairment in short-term spatial memory and long-term spatial memory (object location test and Y maze)
Mishra et al. (2018) [179]	ICV-STZ (Sprague Dawley rats)	Day 11 and Day 18	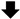 BrdU+Nestin+ cell 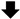 BrdU+NeuN+ cell 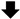 DCX + cell in Day 11 and Day 18 (DG and SVZ)	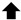 GFAP 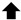 NF-κB in Day 18 (DG and SVZ)	Impairment in learning and memory (Morris water maze test) (Day 18)
Kiyota et al. (2012) [127]	APP+PS1 Tg mice + AAV-mediated expression of IL-10	3 weeks	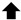 DCX + cell 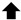 BrdU+NeuN+ cell (SGZ of DG)	IL-10 expression for anti-inflammation reduced gliosis and microglial accumulation around the plaque	N/A
Ghosal et al. (2010) [180]	AICD Tg mice	6 weeks	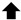 BrdU+DCX+ cell (SGZ of DG)	Anti-inflammation by oral NSAIDs (Ibuprofen and Naproxen)	N/A
Volero et al. (2014) [181]	3xTG-AD	7 weeks	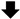 DCX+ cell and synaptic puncta in the dendrites of DCX+ cell	Systemic inflammation by LPS injection	Impairment in spatial memory (Morris water maze test)

Downward arrow: reduced level; Upward arrow: increased level; N/A: not applicable. ICV, intraventricular; STZ, streptozotocin; DCX, doublecortin; BrdU, bromodeoxyuridine; GFAP, glial fibrillary acidic protein; Iba-1, ionized calcium binding adaptor molecule 1; SVZ, subventricular zone; DG, dentate gyrus; Tg, transgenic; NSAIDs, nonsteroidal anti-inflammatory drugs.
